# Understanding tinnitus symptom dynamics and clinical improvement through intensive longitudinal data

**DOI:** 10.1038/s41746-024-01425-w

**Published:** 2025-01-14

**Authors:** Milena Engelke, Jorge Piano Simões, Laura Basso, Nina Wunder, Berthold Langguth, Thomas Probst, Rüdiger Pryss, Winfried Schlee

**Affiliations:** 1 Department of Psychiatry and Psychotherapy, University of Regensburg, Regensburg, Germany; 2 Department of Psychology, Health and Technology, University of Twente, Enschede, The Netherlands; 3 Department of Psychology, Paris Lodron University Salzburg, Salzburg, Austria; 4 Institute of Clinical Epidemiology and Biometry, University of Würzburg, Würzburg, Germany; 5 Institute for Information and Process Management, Eastern Switzerland University of Applied Sciences, St. Gallen, Switzerland

**Keywords:** Diagnosis, Outcomes research

## Abstract

Intensive longitudinal sampling enhances subjective data collection by capturing real-time, dynamic inputs in natural settings, complementing traditional methods. This study evaluates the feasibility of using daily self-reported app data to assess clinical improvement among tinnitus patients undergoing treatment. App data from a multi-center randomized clinical trial were analysed using time-series feature extraction and nested cross-validated ordinal regression with elastic net regulation to predict clinical improvement based on the Clinical Global Impression—Improvement scale (CGI-I). With 50% app compliance (*N* = 129, 8480 entries), the model demonstrated good fit to the test data (McFadden R2 = 0.82) suggesting its generalizability. Clinical improvement was associated with linear declines in tinnitus-related thoughts, jaw tension, tinnitus loudness, increases in happiness, and variability changes in tinnitus loudness and distress. These findings suggest that daily self-reported data on tinnitus symptoms is sensitive to treatment response and provides insights into specific symptom changes that occur during treatment.

## Introduction

Tinnitus, characterized by the perception of an unspecified sound, is a complex and common condition affecting over 740 million adults globally according to recent estimates^1^. In its severe form, it can be accompanied by sleep disturbances, concentration difficulties, memory problems, anxiety and depression, leading to a considerable impairment in quality of life^2,3^. Severe tinnitus is estimated to afflict more than 120 million individuals globally^1^. In European countries, severe tinnitus imposes a profound individual burden with around 1500€ in annual out-of-pocket expenses, as well as socioeconomic burden with approximately 4800€ in annual socioeconomic costs per patient^4^. Efforts have been made to alleviate both health-related and economic burden by the design and implementation of various treatment strategies such as psychological, auditory and pharmacological treatments^5^. Yet, the efficacy of many treatment options remains unclear, which is often attributed to the diverse etiological and clinical manifestation of tinnitus, often referred to as tinnitus heterogeneity^2,5,6^.

However, methodological aspects are often overlooked. In psychosomatic and psychiatric research, treatment outcomes are usually assessed with clinical interviews or self-report measures at selected points in time^7^. Such assessments may fail to capture the dynamic nature of symptoms as they fluctuate over time and across different contexts. Further, patients may struggle to accurately recall the intensity, frequency, and impact of symptomatic episodes, leading to potential underreporting or overreporting of symptoms^8,9^.

Intensive longitudinal sampling of symptoms has thus emerged as a complement to traditional measurement methods^10–12^. A wide range of methods have been framed under the term Experience Sampling or Ecological Momentary Assessment (EMA)^13^. EMA involves (close to) real-time, repeated collection of data in a patient’s natural environment, providing a more granular and immediate perspective on symptomatology^8,13^. Daily or end-of-day diaries (EDD) involve a single assessment per day at fixed intervals, typically in the evening^13^. Although not capturing momentary experiences, EDDs are considered part of the EMA framework due to their repeated administration, which enables a dynamic investigation of the variables of interest^13^. A key advantage of EDDs is their reduced participant burden, however, their retrospective component potentially causes the patient’s end-of-day condition to influence their evaluation of the entire day. Empirical research comparing momentary EMAs throughout the day with EDD data indicated that EDD depicted a more negative picture of certain tinnitus symptoms^14^. However, these effects were small and of uncertain clinical relevance. The authors argue that the slight differences favor the use of EDD over EMA, considering the additional burden imposed by EMA^14^. While EMA is better suited for capturing within-day fluctuations, EDD may be a valid alternative for long-term monitoring. In this manuscript, we adopt the term EMA to encompass a range of designs, including EDD approaches, as outlined in work^13^.

Already 16 years ago, EMA has been discussed as a powerful tool to investigate symptom dynamics and their environmental interactions to enhance understanding of psychopathological mechanisms and treatment response^15^. During the last years, research efforts have strongly increased^16^. With respect to the evaluation of treatment effects, EMA was found to be sensitive to change^17^, to enable a detailed picture of symptom progression^18^, to detect treatment and side effects at an early stage^19,20^, to provide predictive baseline information^21,22^, to reveal interactive effects between symptoms^23^, and to match clinical criterion measures^24–27^.

Despite growing research interest, EMA data should nevertheless be used with caution. Even if momentary or daily self-reports reduce memory biases, EMA answers are likely to be influenced by other cognitive heuristics driven by the current context, assumptions, judgements, comparison standards and interpretation of items^28,29^. Stone and colleagues proposed that EMA and retrospective measurements should not be inherently opposed, but rather, EMA should be viewed as complementary. The choice between these methods should be guided by the theoretical framework underlying the research construct^28^.

Compared to the widespread use of EMA in other health disciplines, the audiological research field is lagging behind^30,31^. Among tinnitus experts, there have been concerns that symptoms could potentially worsen with repeated questioning. Conversely, regular recording of symptom severity might empower patients, giving them more control over their symptoms by better understanding influencing factors^16,32^. Empirical findings have not shown any influence of long-term EMA on tinnitus distress or loudness but revealed the fluctuating nature of tinnitus symptoms and its influence by emotional and environmental factors^33–39^. Further, it has been used to predict the fluctuations of tinnitus by tinnitus-unspecific dimensions such as mood or concentration and the progression of symptoms using neighborhood data^40,41^. Preliminary results regarding clinical utility showed that EMA data unveiled a descriptive decrease in the correlation between tinnitus distress and loudness throughout the duration of an app-based treatment^42^. Further, changes in questionnaire-measured tinnitus distress were associated with trends in EMA-measured tinnitus distress^42^.

Thus, while EMA excels in capturing the ups and downs of daily experiences and has shown initial indications of clinical utility, it remains unclear how clinical improvement manifests within EMA data. To address this question, clinical improvement is operationalized using the patient-reported Clinical Global Impression Scale—Improvement (CGI-I)^43^. The CGI-I is a commonly used 7-point ordinal scale for assessing the subjective degree of change after treatment. It ranges from 1 (very much improved) via 4 (no change) to 7 (very much worse)^44^. In this study, we define clinical improvement as achieving CGI-I scores from 1 to 3, reflecting “very much improved” (1), “much improved” (2) or “minimally improved” (3). Further, feature-based time-series analysis is applied to daily self-reported app data (EMA and EDD questions) which involves extracting meaningful features or characteristics from time-series data^45^. Feature-based time-series analysis has been applied in various domains, including finance, healthcare, environmental monitoring, and manufacturing. In clinical research, features have been extracted from passively generated time-series data, such as movement patterns and vital parameters to predict diagnostic status, change in symptom severity and treatment demands^46–48^.

The primary objective of this study is to identify indicators of daily self-reported tinnitus (Table 1) that characterize clinical improvement. More specifically, we aim to analyze which symptoms change and how these changes manifest through time-series features (Table 2) in patients who respond to treatment. Additionally, we seek to assess the impact of missing app data on the model fit.

**Table 1:** Description of app questions

	Abbreviation	Question (EMA/EDD)	VAS scale [lowest anchor - highest anchor]
1.	t-distress	How burdensome do you find your tinnitus at the moment? (EMA)	not burdensome—very burdensome
2.	t-distress-day	To what extent did you feel affected by the tinnitus today? (EDD)	not at all—the whole day
3.	t-loudness	How loud is your tinnitus at the moment? (EMA)	inaudible—very loud
4.	t-loudness-max	What was the maximum tinnitus volume today? (EDD)	inaudible—very loud
5.	t-thoughts	How often have you thought about tinnitus today? (EDD)	not at all—the whole day
6.	happiness	What emotion would you use to describe today? (EDD)	[sad emoji]—[happy emoji]
7.	jawbone	How tense does your jaw feel right now? (EMA)	not at all tense—very tense
8.	movement	How much did you move today? (EDD)	not at all—very much
9.	neck	How tense does your neck feel right now? (EMA)	not at all tense—very tense
10.	stress	How stressed did you feel today? (EDD)	not at all stressed—very stressed

*Note*. *EMA* Ecological Momentary Assessment question, *EDD* End-of-day diary question. Questions were answered on a continuous visual analogue scale ranging from 0 to 100 (numbers were invisible to the patient).

**Table 2:** Description of time-series features

	Time-series feature	Description
1.	crossing-points	Number of times a time series crosses its median (high values indicate frequent median crossing).
2.	curvature	Curvature of the trend component (STL decomposition). It is based on the coefficient from an orthogonal quadratic regression applied to the trend component (negative values indicate a concave curve, 0 indicates no curvature; positive values indicate a convex curve).
3.	entropy	Forecastability of a time series (Shannon entropy; high values indicate difficulty to forecast).
4.	flat-spots	Number of sections of the data where the series is relatively unchanging (high values indicate constancy).
5.	hurst	Measure of long-term memory of a time series (long-term memory = significant autocorrelations for many lags; high values indicate many autocorrelations).
6.	linearity	Linearity of the trend component (STL decomposition). It is based on the coefficient of a linear regression applied to the trend component (negative values indicate a negative linear trend; 0 indicates no trend; positive values indicate a positive linear trend).
7.	lumpiness	Variance of the variances of non-overlapping windows (length of windows: 10 data points; high values indicate changes in variance).
8.	nonlinearity	Modification of the statistic used in Teräsvirta’s nonlinearity test (large values = nonlinear, values around 0 = linear).
9.	stability	Variance of the means of non-overlapping windows (length of windows: 10 data points; high values indicate changes in mean).
10.	trend	Strength of the trend component (STL decomposition): $1-\frac{{Var}({remainder})}{{Var}\left({remainder}\right)+{Var}({trend})}\left({\rm{high\; values\; indicate\; strength\; trends}}\right)$.

Note. Feature extraction was performed with the theft R package based on the tsfeatures R package^59^. STL decomposition (Seasonal and Trend decomposition using Loess): decomposes a time series in a smoothed trend component, a seasonal component and a remainder component based on a Loess function. Further description of the features: https://cran.r-project.org/web/packages/tsfeatures/vignettes/tsfeatures.html (retrieved on the 29.04.2024)

## Results

### Sample description

**Fig. 1:**
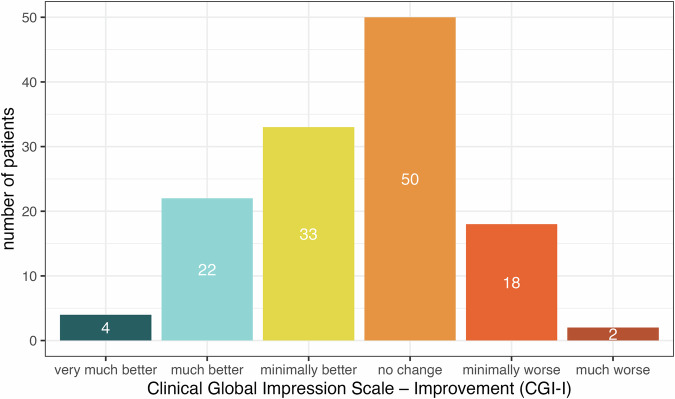
Distribution of CGI-I. *Note*. Distribution of clinical improvement operationalized by the CGI-I. *N* = 129. CGI-I: Clinical Global Impression Scale—Improvement answered on a 7-point Likert scale (1—very much better, 2—much better, 3—minimally better, 4—no change, 5—minimally worse, 6—much worse, 7—very much worse).

The sensitivity analysis revealed that the 50% compliance subsample (corresponding to a minimum of 42 diary entries per patient) yielded the best model fit together with the high compliance subsamples (McFadden pseudo R² = 0.82). In this sample, *N* = 129 patients generated 8480 diary entries. Patients were 47% female, on average 55 years old with a mean tinnitus duration of 132 months and a moderate tinnitus handicap (see Table 3). At final visit, 39% (*N* = 50) indicated no change in their tinnitus complaints compared to before treatment, 26% (*N* = 33) improved minimally, 17% (*N* = 22) indicated good and 3% (*N* = 4) very good treatment response. The remaining patients indicated minimal (14%, *N* = 18) and more severe (2%, *N* = 2) worsening in their tinnitus complaints, while no patient indicated greater deterioration (see Fig. 1; because of rounding the sum does not precisely equal 100%). A comparison of the 50% compliance subsample with the entire RCT sample (*N* = 461) is reported in the Supplementary Material (Supplementary Table 1); patients in the subsample were older (RCT sample: Age [years] = 51.1 ± 12.4 [mean ± SD]).

**Table 3:** Patient characteristics at baseline (*N* = 129)

Sex—no. (%)	
Female	61 (47.3)
Male	68 (52.7)
Age (years)	54.8 ± 12.1
Tinnitus duration (months)	132 ± 117
THI score	48.6 ± 19.6
PHQ-9 score	7.7 ± 5.0
Filled-in diary entries (in %)	78.3 ± 14.0

*Note*. Plus–minus values are means ± SD.

*THI* Tinnitus Handicap Inventory; *PHQ-9* Patient Health Questionnaire for Depression.

### Correlation of clinical improvement with app features

**Fig. 2:**
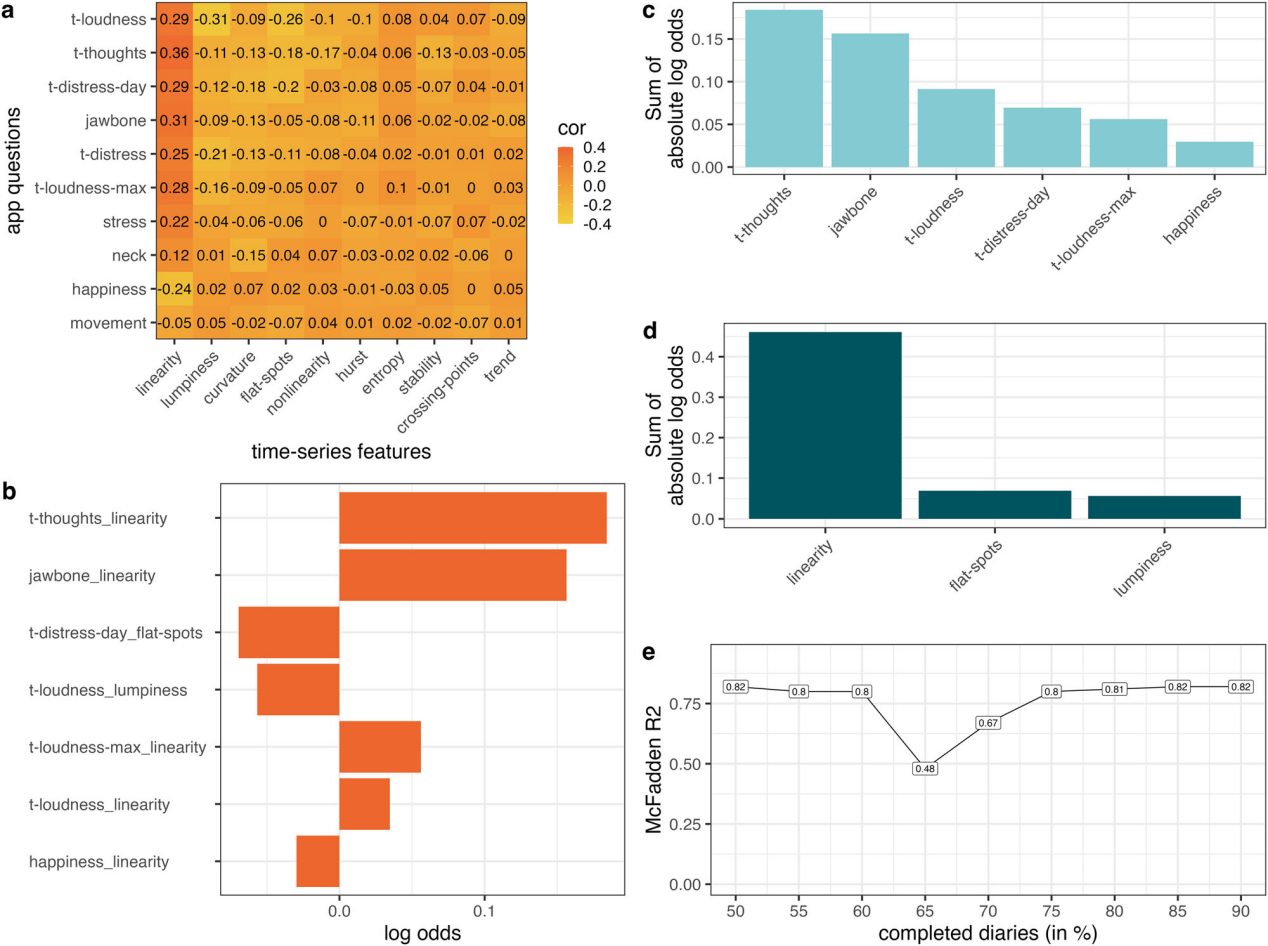
Clinical improvement measured by time-series features of the app questions. *Note*. Clinical improvement measured by time-series features of the app questions. **a** Spearman correlation coefficients between CGI-I and app features (rows and columns are ordered by mean correlation coefficients). **b** Log odds of the ordinal logistic regression with elastic net regularization and 5-fold nested cross-validation. Outcome: CGI-I, Predictors: App features. McFadden R^2^ = 0.82. **c, d** Log odds were derived from an ordinal logistic regression predicting CGI-I by app time-series features. **e** Model fit of predicting CGI-I by app features according to the proportion of completed diary entries. The higher the minimal proportion of completed questionnaires, the smaller the N. Minimum 50% diary entries (*N* = 129), 55% (*N* = 125), 60% (*N* = 113), 65% (*N* = 102), 70% (*N* = 91), 75% (*N* = 75), 80% (*N* = 64), 85% (*N* = 53), 90% (*N* = 37).

After the sample had been identified based on the sensitivity analysis, the association of the app features (time-series features of the app questions) with clinical improvement was explored. Figure 2a illustrates the Spearman correlation between the CGI-I and the 100 app features (rows and columns are ordered by mean correlation coefficients of time-series features and app questions respectively). The top three highest correlation coefficients pertain to linearity of tinnitus-related thoughts (*ρ* = 0.36), linearity of jaw tension (*ρ* = 0.31), and lumpiness of momentary tinnitus loudness (*ρ* = -0.31). Thus, patients indicating clinical improvement had linearly decreasing thoughts about their tinnitus and jaw tension as well as changing fluctuations in tinnitus loudness. The highest mean correlation with the CGI-I was found for momentary tinnitus loudness, tinnitus-related thoughts and daily tinnitus distress among the app questions and for linearity, lumpiness, and curvature among the time-series features.

### Prediction of clinical improvement by app time-series features

Ordinal logistic regression with elastic net penalty was fitted to identify which app questions and time-series features predicted clinical improvement (outcome: CGI-I, predictors: app features). Nested cross validation resulted in an optimal λ of 0.18. This was used to fit the final model which selected 7 out of 100 possible app features (see Fig. 2b, Supplementary Table 2 for the exact coefficients; the remaining features were shrunk to zero). Positive log odds indicate that clinical improvement (i.e. CGI-I values 1–3) is associated with negative values in app time-series features. Negative log odds indicate that clinical improvement is associated with positive values in the respective app time-series feature. Thus, clinical improvement is associated with linearly decreasing tinnitus-related thoughts, jaw tension, maximum tinnitus loudness, and momentary tinnitus loudness. Similarly, clinical improvement is associated with linear increases in happiness. Further, clinical improvement is associated with more flat spots in daily tinnitus distress (i.e. higher constancy) and greater lumpiness in momentary tinnitus loudness (i.e. changing fluctuations) according to the model results (see Table 2 for a description of the features). The model achieved a good fit on the test dataset from nested cross-validation (McFadden R^2^ = 0.82).

### Relevance of app questions and time-series features

To enhance comprehension of the significance of both the app questions and the time-series features in predicting the CGI-I, the sum of the absolute log odds was computed for each app question across the time-series features (see Fig. 2c) and for each time-series feature across the app questions (see Fig. 2d). Among the app questions, the highest predictive relevance for clinical improvement was observed in tinnitus-related thoughts followed by jaw tension, momentary tinnitus loudness, daily tinnitus distress, maximum tinnitus loudness, and happiness. Among the time-series features, linearity was most important for predicting clinical improvement followed by flat-spots and lumpiness. *Linearity* is the fluctuation-corrected linearity of the trend, with negative values indicating negative linear trends and positive values indicating positive linear trends. *Flat-spots* is an indicator of constancy, higher values indicate more constancy. *Lumpiness* is an indicator of the change in variance, high values indicate more changes in variance (could be both more or less variance).

### Sensitivity analysis

Figure 2e illustrates the model fit, as assessed by McFadden R^2^, for models with varying compliance rates. McFadden R^2^ remained approximately constant around 0.8 for compliance rates ranging from 50% to 90% of app entries with a drop at 65% and 70%. The coefficients of the remaining models are reported in the Supplementary Fig. 1-8. Higher lambda values shrink the coefficients stronger which leads to smaller odds ratios and smaller selection of features. Those models are less prone to overfitting and demonstrate better fit to the data, as indicated by higher McFadden R^2^ on the test sets. Certain features remain stable across the models with different compliance rates (e.g., t-thoughts_linearity and jawbone_linearity). Notably, due to the small sample size, subsamples with higher compliance rates could not be tested.

## Discussion

Our findings underline the feasibility of using daily self-reported symptoms to measure clinical improvement in tinnitus patients undergoing treatment which is in line with findings from other health research fields^12,16,26^.

We were able to show how treatment response is reflected longitudinally in tinnitus symptoms. Based on our regression model, patients were more likely to improve if they had a linear decline in tinnitus-related thoughts, jaw tension, maximum tinnitus loudness, and momentary tinnitus loudness, a linear increase in happiness, as well as constancy in daily tinnitus distress and a change in fluctuation in momentary tinnitus loudness. The model showed a good fit to the test data, suggesting its generalisability. The results are mostly, but not completely consistent with the correlation analysis which is probably caused by collinearity issues that have been addressed by the elastic net algorithm. Notably, the correlation coefficient of flat spots in daily tinnitus distress with the CGI-I is the smallest among the selected features (ρ = -0.2).

The most predictive time-series feature for clinical improvement was linearity which is the linearity of the smoothed trend component corrected from high-frequent fluctuations such as noise and seasonality. Thus, not the magnitude of symptom reduction, but the linearity of the trend was relevant. This suggests that patients may prefer steady progress, with high fluctuations disrupting their sense of improvement. Additionally, though less influential, features such as flat spots and lumpiness were also linked to improvement. Flat spots in tinnitus distress, which indicates constancy, was positively associated with improvement, suggesting that constancy in tinnitus distress is experienced as more beneficial than fluctuations in tinnitus distress. In contrast, lumpiness in tinnitus loudness, which reflects variability in fluctuations, was positively associated with improvement, suggesting that more variability in loudness fluctuation predicts a beneficial outcome. The joint consideration of the other features suggests that a strong fluctuation of symptoms during treatment is unfavourable for treatment response. Thus, we hypothesize that variability in loudness fluctuation unfolds as a reduction in loudness fluctuation. However, it is not specified by the feature and requires further investigation. These findings align with an earlier study investigating the feasibility of using EMA to detect treatment effects in fibromyalgia patients. They found that next to a decline in average pain, a reduction in pain variability contributed incrementally to clinical improvement^26^.

Looking at which symptoms change, most predictive for clinical improvement were changes in tinnitus-related thoughts, jaw tension, momentary tinnitus loudness, daily tinnitus distress, maximum tinnitus loudness, and happiness (relevance in descending order). Those symptoms that are usually applied as outcome measures^49^, i.e. tinnitus distress and loudness, are accompanied by psychological and somatic symptoms. This finding adds an interesting dimension to the understanding of clinical improvement in tinnitus, as it underscores the importance of considering general health symptoms alongside tinnitus-specific indicators. While the main mechanism of established tinnitus therapies typically involves reducing tinnitus-related distress, it is noteworthy that patients may prioritize loudness reduction or complete relief from tinnitus^50^. Thus, the implementation of patient-centered, personalized EMA, wherein clinically relevant symptoms are identified by the patient and assessments are tailored accordingly, holds promise for enhancing clinical diagnostics and care^32^.

The sensitivity analysis demonstrated the stability of key predictors (e.g. t-thoughts_linearity and jawbone_linearity) as well as consistent model fit across varying compliance rates. McFadden R^2^ remained stable around 0.8 for compliance rates of 50–60% and 75–90%, suggesting robust modeling of clinical improvement using EMA data even in less compliant samples, which often reflect real-world scenarios. However, models with compliance rates of 65% and 70% appeared overfitted, as evidenced by smaller penalization factors (lambda), larger coefficients, and lower fit on the test set. It is important to note that the overall compliance in our study was lower than the typical 79% seen in pure EMA studies^51^, likely because the daily diary was a secondary aspect of the clinical trial. A comparison of the 50%-compliance subsample with the entire RCT sample revealed variation only in age, while other baseline characteristics and CGI-I distributions were comparable. Although our findings highlight the feasibility of obtaining robust models from less compliant samples, efforts to increase compliance rates in EMA remain essential. Higher compliance not only improves data quality and statistical power but also enhances representativeness of the sample. EMA experts recommend strategies like improving instructions, encouraging questions, practice, and providing feedback to enhance both compliance and data quality^28^.

The following aspects should be taken into account when evaluating the results. The CGI-I was used to assess clinical improvement which asks patients to evaluate the overall impact of the intervention on their tinnitus. This method has both benefits and limitations. It values patients’ self-assessment, treating them as experts on their own condition. However, its retrospective nature relies on patients recalling their pre-therapy state, which can introduce biases. Incorporating clinician-rated assessments alongside patient self-reports could provide a more balanced evaluation, capturing both subjective experiences and clinical evaluations^52^. Nevertheless, the “true” measure of treatment efficacy remains elusive in subjective assessments, highlighting the need for a biomarker to gauge the severity of the condition. Further, the features were pre-selected based on an emphasis on capturing the dynamics of change and based on the relevant literature^47,48^; a different feature selection could have led to different results. We acknowledge that the use of a linear predictive model, such as logistic regression, may influence feature selection, favoring features with clear linear relationships to the outcome. However, the selected features align with clinically meaningful patterns of improvement. Also, EMA questionnaires need to be carefully evaluated in terms of their clinimetric properties (psychometric criteria transferred to clinical outcome measures). This includes sensitivity (e.g., ability to detect treatment effects, ability to differentiate active treatment from placebo), clinical validity (ability to discriminate between subjects with and without a condition e.g. using cutoff scores), predictive validity (ability to predict treatment response and clinical outcomes), and incremental validity (each item provides distinctive clinical information)^53^. Additionally, there is a need for discussion whether clinimetric criteria should be updated to accommodate EMA measures, given that these criteria were originally introduced for traditional questionnaires.

In summary, we provided evidence that EMA is sensitive for change across a range of tinnitus treatments. By longitudinally drawing on the symptom level, EMA was able to show which symptoms change and how they are changing if patients respond to treatment. The latter was realised by the application of time-series features, a computational method that characterizes dynamics of time-series which are not visible to the eye. This allows for an easily applicable, understandable and effective utilization of longitudinal data to understand time dynamics without compromising valuable information or imputing missing data^45^. In comparison to questionnaires that often rely on total scores^10^, the use of EMA data not only allows treatment effects to be described at the symptom level, but could facilitate an exploratory investigation into which specific symptoms are impacted by a particular treatment and are thus paving the path towards personalized treatments. Finally, questionnaires depict only few snapshots in time which limits their ability to measure the longitudinal progress of symptoms^27^. This study demonstrated the complexity of symptom courses during treatment and the importance of longitudinal data, as linearity and fluctuations were identified to be predictive for treatment success.

Our findings might stimulate both future research and clinical practice in tinnitus. We encourage researchers to replicate our findings and expand them, e.g. by investigating individual differences, early treatment response or different feature sets. Importantly, we need to explore mechanisms to substantially improve compliance towards EMA protocols within clinical studies. Further, while passive sensing has been explored concerning the prediction of clinical status and change^47,48^, efforts could be made to investigate whether combining EMA with passive sensing enhances the prediction and understanding of treatment response^12,54^. In clinics, EMA could offer a dynamic approach to monitoring patient progress in real-time and reduce costs by the reduction of in-person visits. Clinicians can incorporate EMA techniques into routine clinical assessments, allowing for ongoing tracking of key variables like general stress levels, tinnitus distress, and emotional states. This enables timely adjustments to treatment plans based on individual patient needs and fluctuations in symptoms.

## Methods

Data were drawn from a multi-center parallel-arm randomized clinical trial that sought to examine the effect of single and combination treatments in patients with chronic subjective tinnitus^55,56^. The study was registered at https://clinicaltrials.gov/study/NCT04663828. All participants gave written informed consent and ethics approval was obtained from the ethical committees of all five trial sites (University of Regensburg, Regensburg, Germany [combined ethical approval for clinical sites Berlin and Regensburg]; Katholieke Universiteit Leuven, Leuven, Belgium; Ethniko Kai Kapodistriako Panepistimo Athinon, Athens, Greece; Hospital Universitario Virgen de las Nieves/ Hospital Clinico Universitario San Cecilio, Granada, Spain).

### Participants

Patients were included if they reported having chronic tinnitus ( ≥ 6 months) as their primary complaint, had at least mild tinnitus handicap (Tinnitus Handicap Inventory [THI] ≥ 18), were aged between 18 and 80 years, and had not started any other tinnitus-related treatments in the last 3 months before study start. Patients with objective tinnitus, otosclerosis, acoustic neuroma, acute ear infections, Meniere’s disease, severe hearing loss, as well as serious internal, neurological, or psychiatric conditions were excluded.

### Study Design and Procedure

The data were collected between April 2021 and December 2022 at five clinical sites across Europe. Participants were randomized to one out of ten treatment arms, which were either single treatments (Cognitive Behavioural Therapy [CBT], Hearing Aids [HA], Structured Counseling [SC], Sound Therapy [ST]) or combinations thereof (CBT + HA, CBT + SC, CBT + ST, HA + SC, HA + ST, SC + ST) lasting for 12 weeks. Demographic and clinical characteristics were assessed at baseline (before treatment), interim visit (during treatment after 6 weeks), final visit (after 12 weeks of treatment) and follow-up (after 36 weeks). During the 12-week treatment phase, self-reported tinnitus symptoms were further gathered daily using an end-of-day electronic diary via the UNITI smartphone application^57^. Additional information about the trial is described elsewhere^55^.

### Measures

The Clinical Global Impression Scale—Improvement (CGI-I)^43^ assessed at final visit served as the criterion measure of clinical improvement. In a single query, patients are prompted to assess the overall amelioration of their tinnitus symptoms in relation to the period preceding treatment (“Please rate the total improvement of your tinnitus complaints compared to before beginning of treatment.”) using a 7-point Likert scale (1—very much better, 2—much better, 3—minimally better, 4—no change, 5—minimally worse, 6—much worse, 7—very much worse). The validity of the CGI-I as an outcome measure has been examined and it was shown to be sensitive to change^58^.

Daily self-reported data on tinnitus symptoms was assessed via an end-of-day electronic diary in the UNITI smartphone application, which prompted the patient every evening at 19:30 to rate several EMA and EDD questions on a continuous visual analogue scale (VAS; questions listed in Table 1). EMA questions aimed to assess the condition of the respective moment, while EDD questions were intended to reflect on the whole day. Patients were asked to answer the questions (EMA and EDD) once every day over the 12-week treatment phase, so ideally 84 answered app entries were expected per patient. The assessment could only be submitted once all questions had been answered.

### Statistical analysis

Before extracting time-series features from app data, the range of diary answers per patient was calculated. A requirement for meaningful feature extraction and analysis was minimal variability in all app questions, thus, *N* = 263 patients’ time series were eligible for feature extraction (RCT sample size *N* = 461). We selected 10 features out of a pool of six open-source time-series feature sets (included in the R package *theft*^59^). On one hand, feature selection was based on capturing the dynamics of change as it was expected that symptoms would change as a result of the clinical intervention. On the other hand, the selection was based on citations in the clinical feature-based time-series literature to identify the features that have already been shown to be relevant (see Table 2 for an overview and description of the selected features)^47,48^. Each patient generated 10 time-series (10 app questions), thus, extraction of 10 time-series features led to 100 features describing each patient (10 app questions [Table 1] x 10 features [Table 2]). There was no imputation of missing data before feature extraction.

In the second step, we performed a sensitivity analysis to identify the optimal amount of completed diary entries to maximize the model fit of predicting treatment response in our dataset. Due to real-world data usage, the higher the compliance rate, the smaller the sample size available for analysis. We fitted ordinal logistic regression models with a nested cross-validated elastic net penalty for minimal compliance rates between 50% and 90% in 5% steps (i.e., 9 regression models; results are reported in the Supplementary Fig. 1-8). Due to the high number of potentially correlated predictors, elastic net was chosen to perform variable selection and shrinkage. CGI-I at final visit was used as outcome variable. Predictors included the 100 scaled times-series features which have been described above. To minimize overfitting, 5-fold nested cross-validation was performed to tune the elastic net across 20 different lambda values (λ) based on the best AIC fit (R package *ordinalNet*, function *ordinalNetCV*, method is based on example 5 in work^60^). The nested cross-validation procedure enables a performance evaluation of the tuned model on data that has not been used to train the model. First, the data is split into five folds, each time leaving out the test dataset. Ordinal elastic net regression (α = 0.5) is performed on each of the five training datasets on a sequence of 20 λ values (default settings; λ_max_ equals the smallest value that sets every coefficient to zero). The best AIC fit is selected on each fold and the out-of-sample prediction is then assessed on the hold-out test set. The λ value with the best out-of-sample log-likelihood of the five folds is used in the final model to obtain the coefficient estimates. The performance measures on the test set are taken to obtain model fit. This was determined by McFadden pseudo R² that compares the log-likelihood of the full model with the log-likelihood of the null model (intercept-only model)^61^

In the third step, the sample with the compliance rate demonstrating the best prediction was subject to further investigation. Spearman correlation coefficients were computed between CGI-I and time-series app features. This served as a first description of the association without reporting any *p*-values, therefore correction for multiple testing was not applied. The cumulative model was fitted reversed to ensure consistent sign between correlation and regression results (cumulative probabilities *P*(*Y* ≥ 2), …, *P*(*Y* ≥ *K* + 1); with *K* + 1 response categories). The coefficients of the ordinal logistic regression are displayed as log odds, which is the logarithm of the odds ratio (probability of success/probability of failure). A positive coefficient means that as the predictor variable increases by one unit, the log odds of being in a higher category (less clinical improvement) increase. In order to deconstruct the results obtained from the regression analysis and identify the relevance of both time-series features and app questions in measuring clinical improvement, absolute values of the log odds were summed for the respective app questions across the time series features and vice versa. All analyses were performed in R (version 4.2.2).

## Supplementary information

Supplemental Material

## Data Availability

The dataset for this study is available from the corresponding author upon request.

## Code availability

The underlying code for this study is available from the corresponding author upon request.
