# Supplementary material for: Understanding tinnitus symptom dynamics and clinical improvement through intensive longitudinal data
Source: NPJ Digit Med. 2025 Jan 14;8:27. doi: 10.1038/s41746-024-01425-w (PMC11733005; doi:10.1038/s41746-024-01425-w)
Supplement: Supplementary file 1 — Supplemental Material [file 41746_2024_1425_MOESM1_ESM.pdf]

Supplementary Table 1. Comparison of 50% app compliance subsample with entire RCT sample

	50% compliance (N = 129)	RCT (N = 461)	p value
**Sex**			
female	61 (47.3%)	190 (41.2%)	0.258
male	68 (52.7%)	271 (58.8%)	
**Age (years)**			
Mean (SD)	54.8 (12.1)	51.1 (12.4)	0.002
Median [Min, Max]	56.0 [23.0, 77.0]	53.0 [19.0, 77.0]	
**Tinnitus duration (months)**			
Mean (SD)	132 (117)	119 (113)	0.31
Median [Min, Max]	105 [6.00, 480]	84.0 [6.00, 480]	
**THI score baseline**			
Mean (SD)	48.6 (19.6)	48.0 (19.7)	0.738
Median [Min, Max]	46.0 [18.0, 94.0]	46.0 [8.00, 96.0]	
**TFI score baseline**			
Mean (SD)	51.5 (21.2)	48.6 (20.3)	0.159
Median [Min, Max]	54.0 [4.00, 94.0]	49.0 [2.00, 95.0]	
**PHQ-9 score baseline**			
Mean (SD)	7.73 (5.02)	7.32 (4.87)	0.42
Median [Min, Max]	7.00 [0, 23.0]	7.00 [0, 27.0]	
**CGI-I score final visit**			
very much better	4 (3.1%)	16 (3.5%)	0.854
much better	22 (17.1%)	56 (12.1%)	
minimally better	33 (25.6%)	112 (24.3%)	
no change	50 (38.8%)	132 (28.6%)	
minimally worse	18 (14.0%)	43 (9.3%)	
much worse	2 (1.6%)	5 (1.1%)	

Note. Plus–minus values are means ± SD.

THI = Tinnitus Handicap Inventory; TFI = Tinnitus Functional Index; PHQ-9 = Patient Health Questionnaire for Depression; CGI-I = Clinical Global Impression Scale – Improvement. Final visit: 12 weeks after baseline (treatment end).

Supplementary Table 2. Coefficients of the main model

Feature	Log odds	Odds ratio
t-thoughts_linearity	0.18	1.20
jawbone_linearity	0.16	1.17
t-distress-day_flatspots	-0.07	0.93
t-loudness_lumpiness	-0.06	0.95
t-loudness-max_linearity	0.06	1.06
t-loudness_linearity	0.03	1.04
happiness_linearity	-0.03	0.97

Note. Log odds of the ordinal logistic regression with elastic net regularization and 5-fold cross-validation. Outcome: CGI-I, Predictors: EMA times-series features.

Supplementary Figure 1. Model results with minimal 55% completion rate (N = 125).

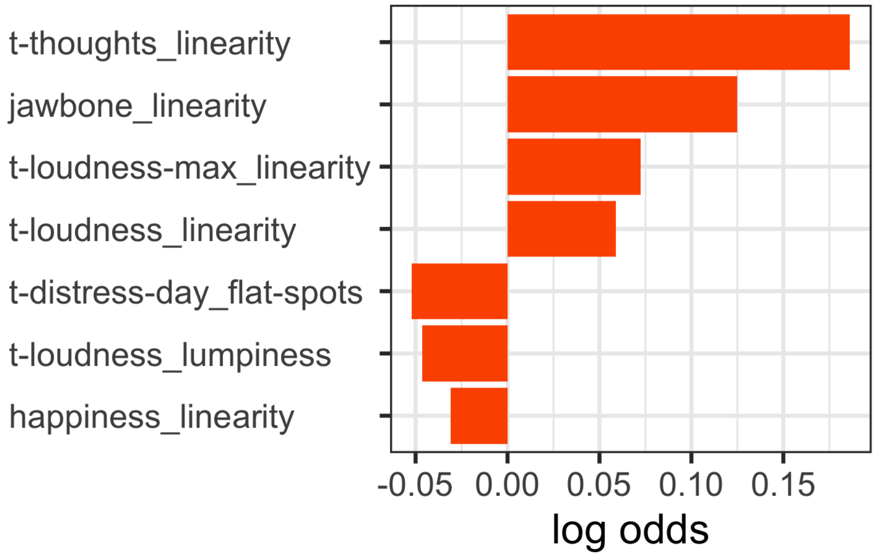

Note. N = 125, Lambda = 0.19, Loglik = -36.4. Log odds of the ordinal logistic regression with elastic net regularization and 5-fold cross-validation. Outcome: CGI-I, Predictors: EMA times-series features.

Supplementary Figure 2. Model results with minimal 60% completion rate (N = 113).

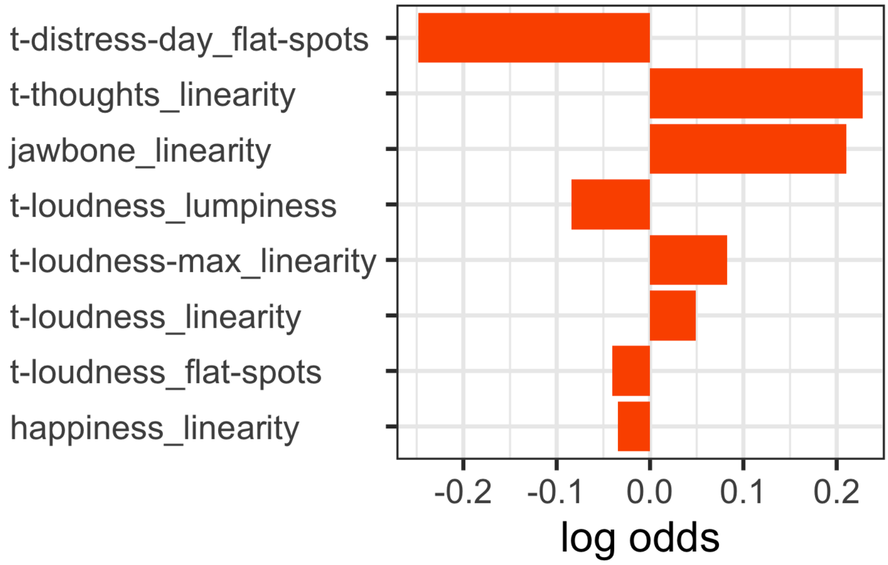

Note. N = 113, Lambda = 0.15, Loglik = -30.8. Log odds of the ordinal logistic regression with elastic net regularization and 5-fold cross-validation. Outcome: CGI-I, Predictors: EMA times-series features.

Supplementary Figure 3. Model results with minimal 65% completion rate (N = 102).

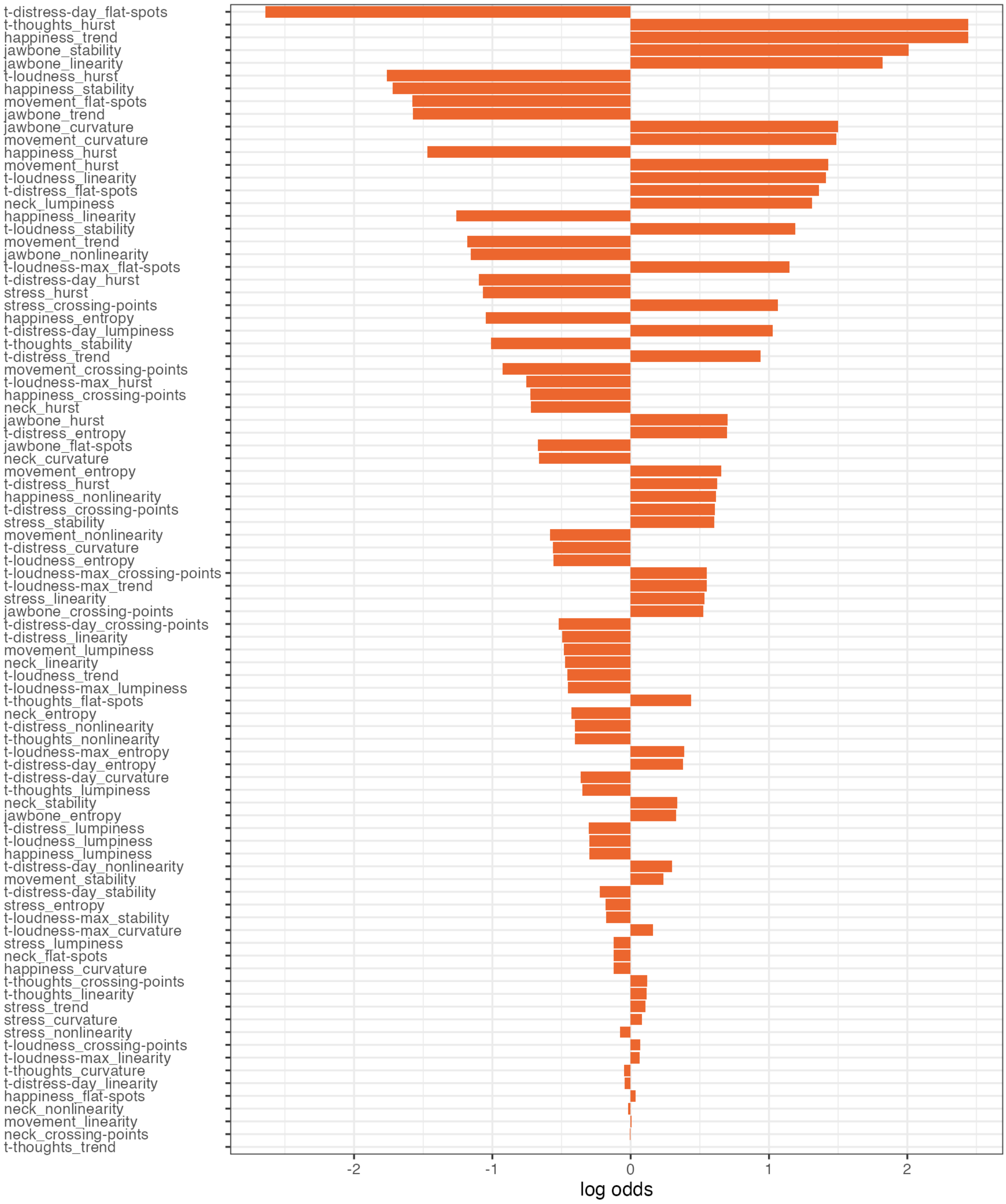

Note. N = 102, Lambda = 0.004, Loglik = -74.8. Log odds of the ordinal logistic regression with elastic net regularization and 5-fold cross-validation. Outcome: CGI-I, Predictors: EMA times-series features.

Supplementary Figure 4. Model results with minimal 70% completion rate (N = 91).

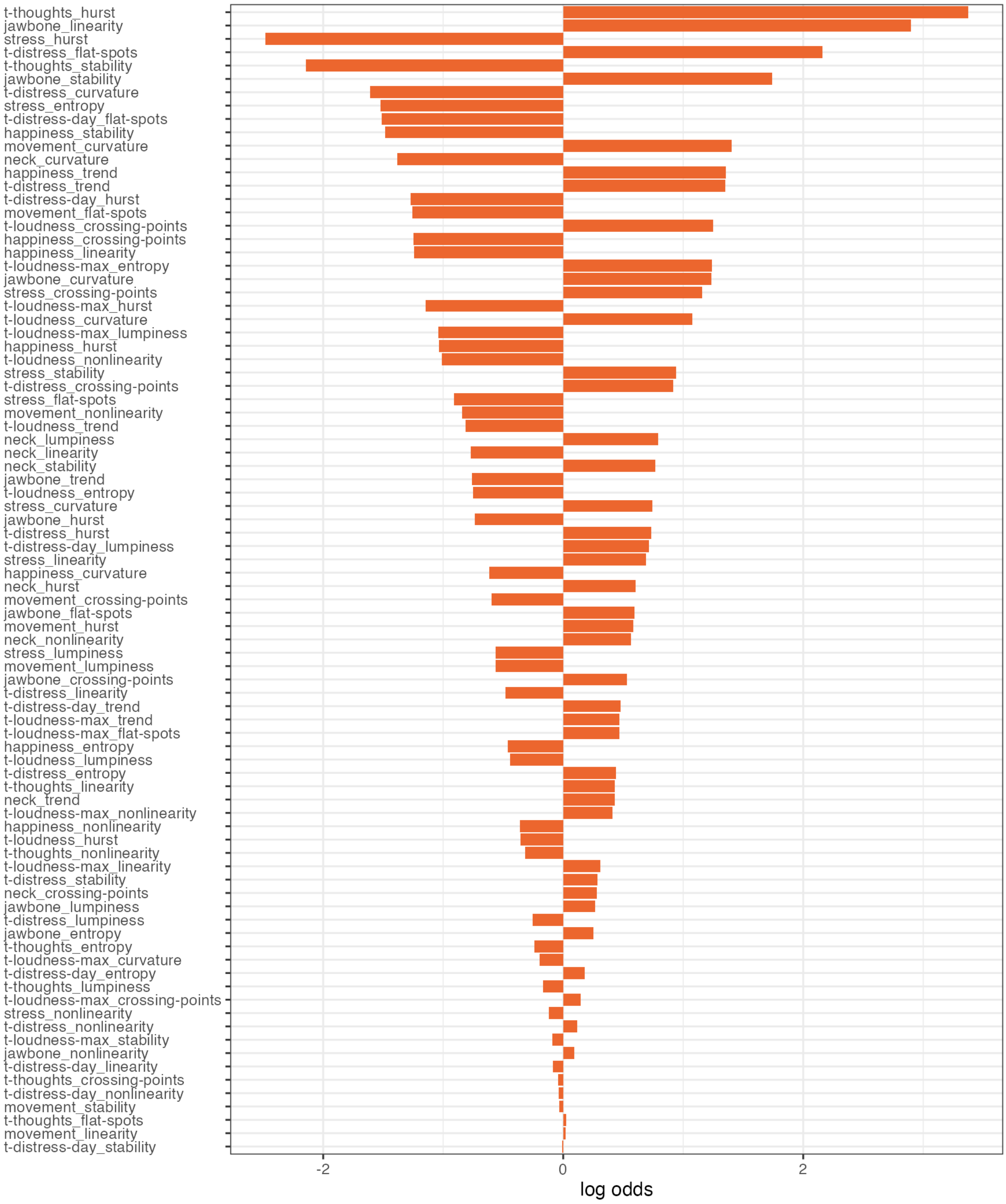

Note. N = 91, Lambda = 0.04, Loglik = -41.3. Log odds of the ordinal logistic regression with elastic net regularization and 5-fold cross-validation. Outcome: CGI-I, Predictors: EMA times-series features.

Supplementary Figure 5. Model results with minimal 75% completion rate (N = 75).

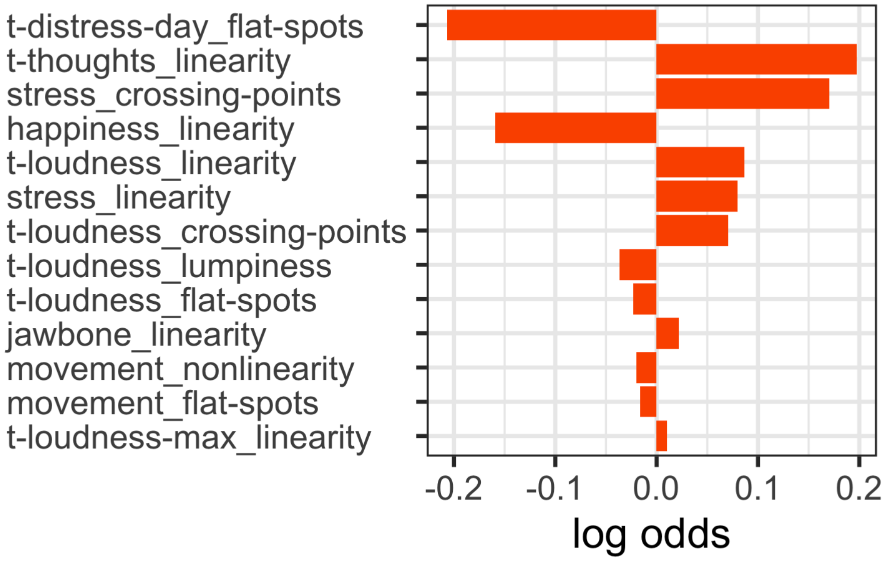

Note. N = 75, Lambda = 0.19, Loglik = -21.2. Log odds of the ordinal logistic regression with elastic net regularization and 5-fold cross-validation. Outcome: CGI-I, Predictors: EMA times-series features.

Supplementary Figure 6. Model results with minimal 80% completion rate (N = 64).

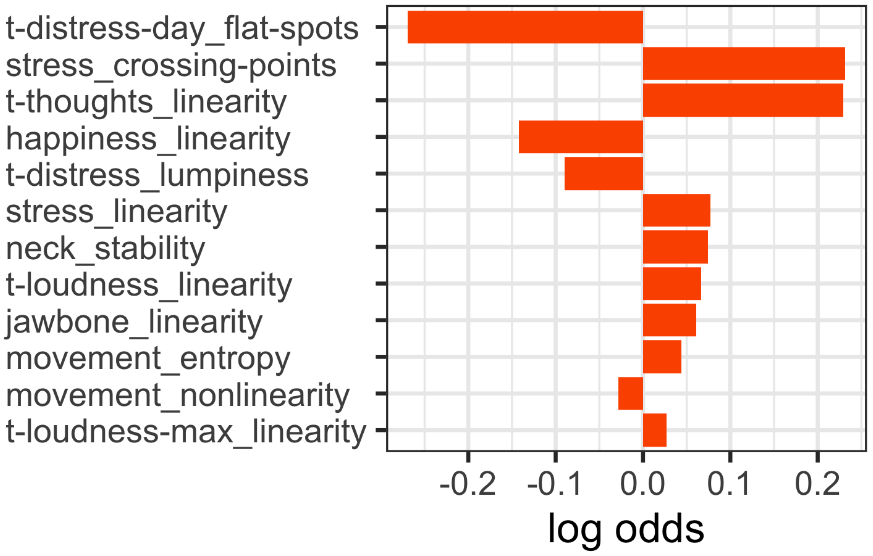

Note. N = 64, Lambda = 0.19, Loglik = -16.0. Log odds of the ordinal logistic regression with elastic net regularization and 5-fold cross-validation. Outcome: CGI-I, Predictors: EMA times-series features.

Supplementary Figure 7. Model results with minimal 85% completion rate (N = 53).

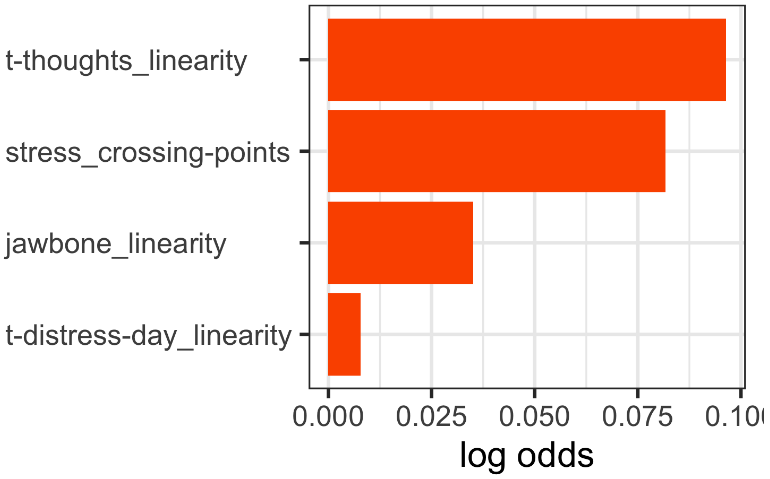

Note. N = 53, Lambda = 0.37, Loglik = -12.9. Log odds of the ordinal logistic regression with elastic net regularization and 5-fold cross-validation. Outcome: CGI-I, Predictors: EMA times-series features.

Supplementary Figure 8. Model results with minimal 90% completion rate (N = 37).

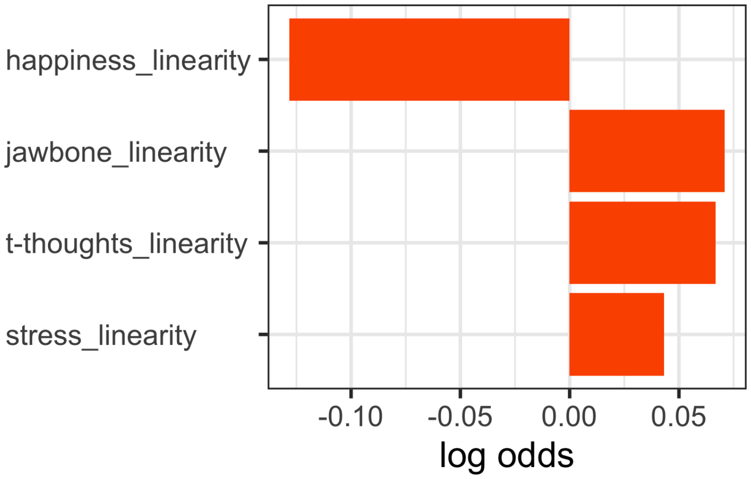

Note. N = 37, Lambda = 0.51, Loglik = -9.0. Log odds of the ordinal logistic regression with elastic net regularization and 5-fold cross-validation. Outcome: CGI-I, Predictors: EMA times-series features.
